# Bis(2-hy­droxy-2,3-di­hydro-1*H*-inden-1-aminium) tetra­chlorido­palladate(II) hemihydrate

**DOI:** 10.1107/S2414314624000592

**Published:** 2024-01-26

**Authors:** Ovender Singh, Jungwi Mok, Hyoung-Ryun Park, Junseong Lee

**Affiliations:** a Department of Chemistry, Chonnam National University, Gwangju, Republic of Korea; Sunway University, Malaysia

**Keywords:** crystal structure, hydrogen bonding, palladium tetra­chloride, amino­indanol

## Abstract

A new square-planar palladium complex salt hydrate, (C_9_H_12_NO)_2_[PdCl_4_]·0.5H_2_O, has been characterized. The asymmetric unit of the complex salt comprises two [PdCl_4_]^2−^ dianions, four 2-hy­droxy-2,3-di­hydro-1*H*-inden-1-aminium cations, each derived from (1*R*,2*S*)-(+)-1-amino­indan-2-ol, and one water mol­ecule of crystallization. In the crystal, a two-dimensional layer parallel to (001) features a number of O—H⋯O, N—H⋯O, O—H⋯Cl and N—H⋯Cl hydrogen bonds.

## Abstract 2

In the title salt hydrate, (C_9_H_12_NO)_2_[PdCl_4_]·0.5H_2_O, the Pd^II^ atom is coordinated by four chloride anions and the charge balance is provided by two ammonium cations formed from (1*R*,2*S*)-(+)-1-amino­indan-2-ol.

## Graphical abstract

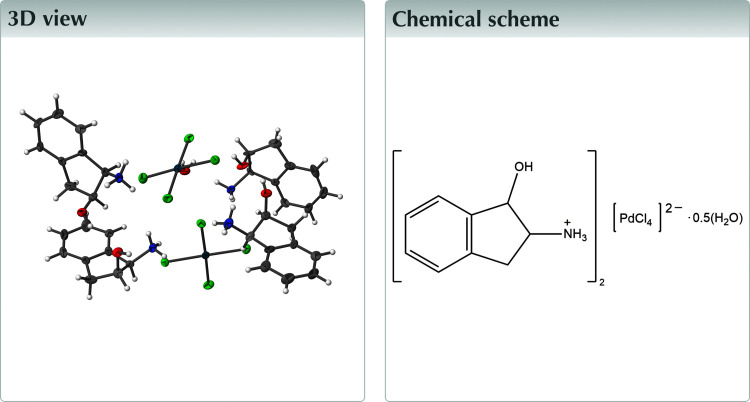

## Structure description

Palladium catalysis has become a versatile tool in modern organic synthesis, revolution­izing chemical transformations (Chen *et al.*, 2013 ▸; Biffis *et al.*, 2018 ▸; Han, 2023 ▸). In particular, palladium complexes with chiral ligands has received increasing attention in asymmetric reactions (Uchikura *et al.*, 2023 ▸). In this field, we have been inter­ested in the synthesis of chiral palladium complexes and recently reported several palladium complexes with chiral tridentate ligands based on (1*R*,2*S*)-(+)-1-amino­indan-2-ol (Singh *et al.*, 2022 ▸). During these studies, we unexpectedly isolated the title complex, (C_9_H_12_NO)_2_[PdCl_4_]·0.5H_2_O. In the complex, the Pd^II^ centre did not form the anti­cipated bonds to O and N atoms of the ammonium chloride salt based on (1*R*,2*S*)-(+)-1-amino­indan-2-ol, but binds with chloride ions instead, to form a [PdCl_4_]^2−^ dianion.

**Figure 1:**
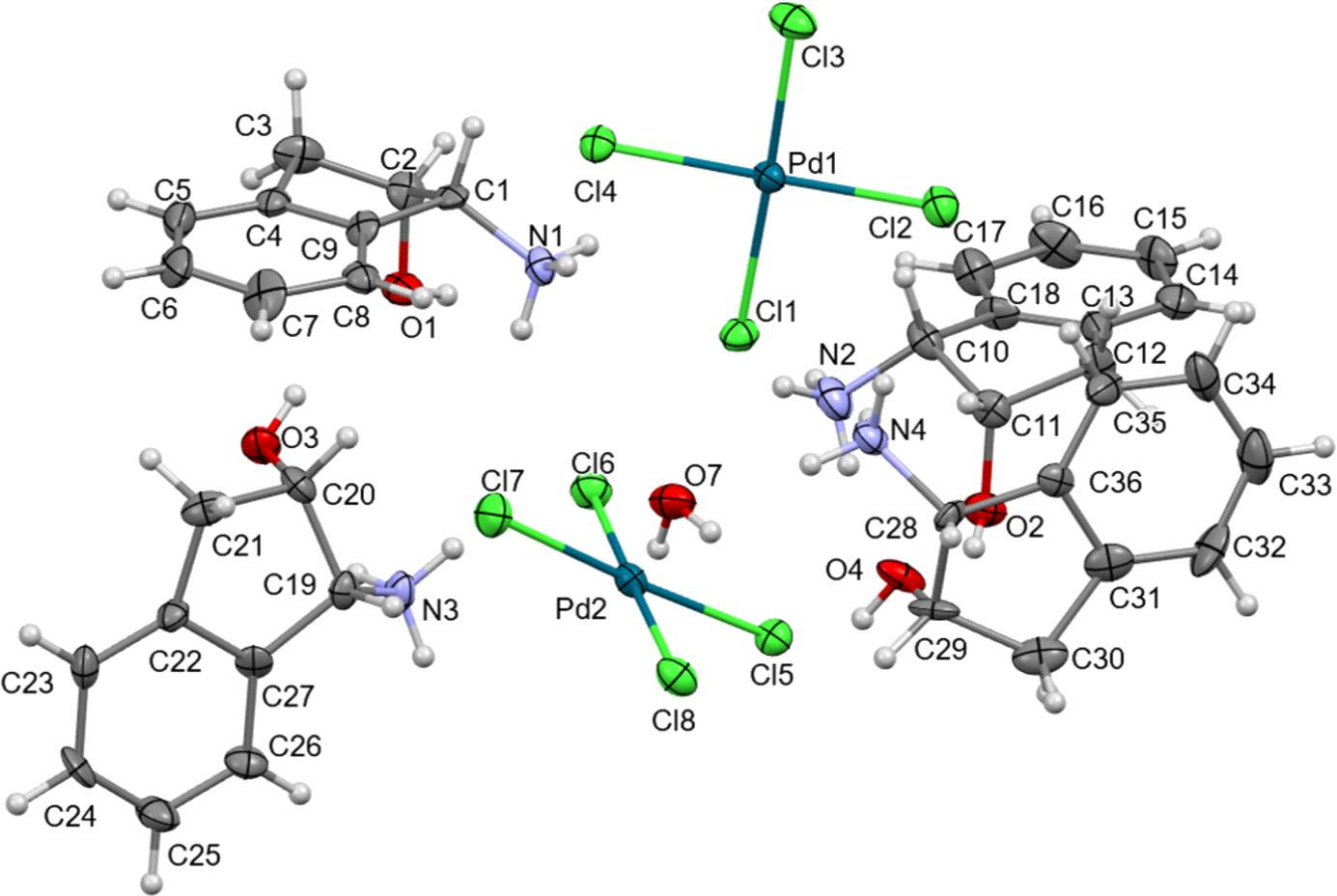
The mol­ecular structures of the components comprising the asymmetric unit of the title complex salt hydrate, showing the atom-numbering scheme and displacement ellipsoids at the 50% probability level.

The asymmetric unit comprises two [PdCl_4_]^2−^ dianions, four ammonium cations derived from (1*R*,2*S*)-(+)-1-amino­indan-2-ol and a H_2_O mol­ecule of crystallization, as shown in Fig. 1 ▸. The dianions adopt a square-planar Pd^II^ coordination environment. A search of the Cambridge Structural Database (CSD, Version 5.42, November 2020; Groom *et al.*, 2016 ▸) provided a large number of related tetra­chlorido- and tetra­bromido­palladate salts (*e.g.* Mais *et al.*, 1972 ▸; Martin *et al.*, 1975 ▸; Takazawa *et al.*, 1988 ▸).

**Figure 2:**
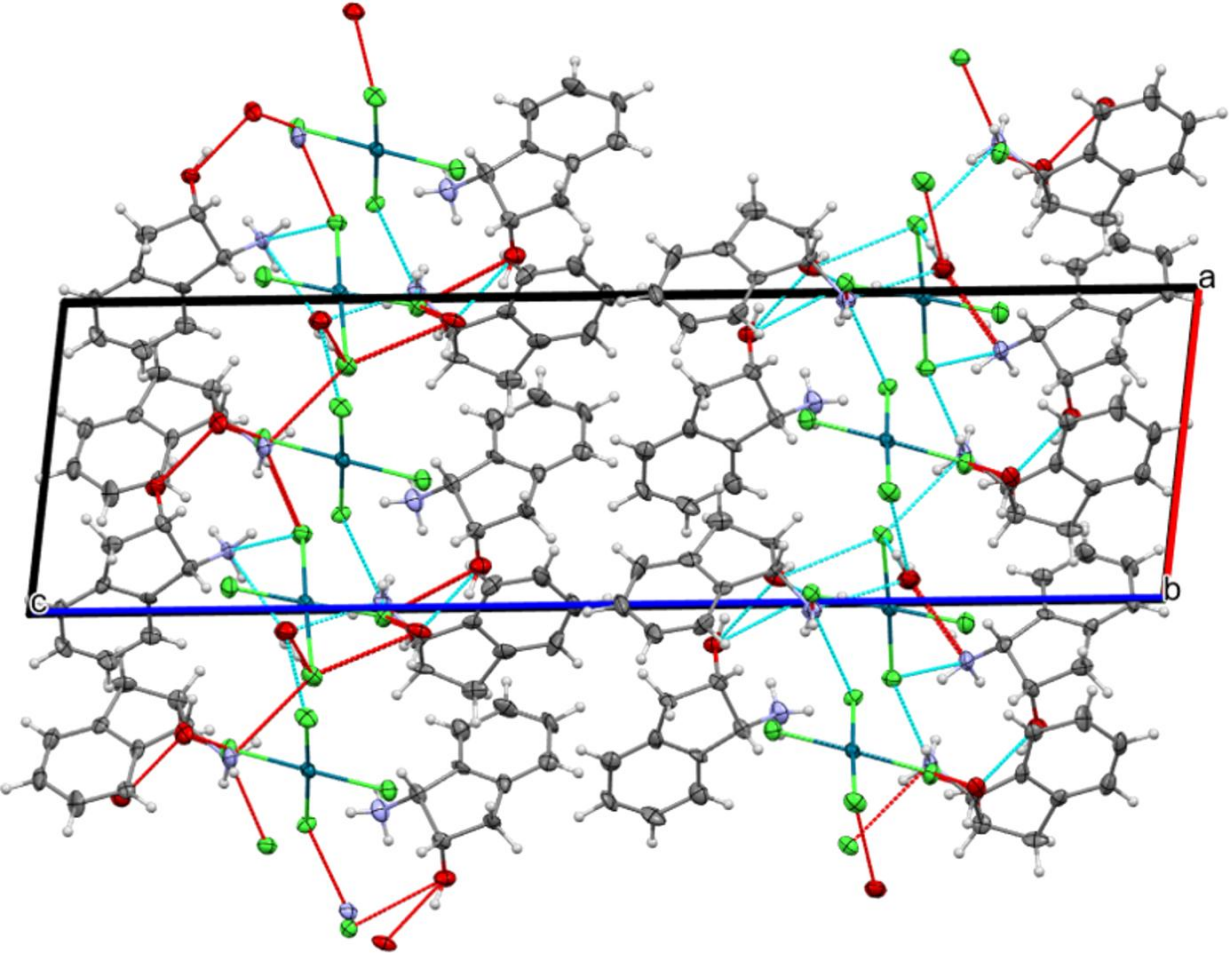
The packing of the title complex salt hydrate in projection along the *b* axis. The dashed lines indicate inter­molecular hydrogen bonds. All H atoms not involved in hydrogen bonding have been omitted for clarity and displacement ellipsoids are drawn at the 50% probability level.

In the packing, a number of O—H⋯O, N—H⋯O, O—H⋯Cl and N—H⋯Cl hydrogen bonds are observed (Table 1 ▸). All O and N atoms participate in hydrogen bonding, but not all Cl atoms. The hydrogen bonds feature within a two-dimensional layer structure parallel to (001) (Fig. 2 ▸).

## Synthesis and crystallization

Palladium(II) chloride (0.089 g, 0.502 mmol) was added to a methanol (10 ml) solution of (1*R*,2*S*)-(+)-1-amino­indan-2-ol (0.149 g, 1.00 mmol) in the presence of aqueous HCl (1 *M*, 1 ml). The resulting solution was heated at 303 K for 12 h and filtered through a 0.45 mm PTFE syringe filter. Crystals suitable for X-ray diffraction studies were obtained by slow evaporation of a saturated methanol solution of the salt hydrate at 298 K.

## Refinement

Crystal data, data collection and structure refinement details are summarized in Table 2 ▸. Owing to poor agreement, 17 reflections were omitted from the final cycles of refinement; see CIF for details.

## Supplementary Material

Crystal structure: contains datablock(s) I. DOI: 10.1107/S2414314624000592/tk4099sup1.cif

Structure factors: contains datablock(s) I. DOI: 10.1107/S2414314624000592/tk4099Isup2.hkl

Supporting information file. DOI: 10.1107/S2414314624000592/tk4099Isup3.cdx

CCDC reference: 2327302

Additional supporting information:  crystallographic information; 3D view; checkCIF report

## Figures and Tables

**Table 1:** Hydrogen-bond geometry (Å, °)

*D*—H⋯*A*	*D*—H	H⋯*A*	*D*⋯*A*	*D*—H⋯*A*
O1—H1⋯Cl4^i^	0.82	2.57	3.116 (9)	126
N1—H1*B*⋯Cl6	0.89	2.43	3.177 (10)	141
O2—H2*A*⋯O4	0.82	2.13	2.870 (11)	150
O3—H3⋯O1	0.82	2.01	2.779 (11)	155
N3—H3*C*⋯Cl6	0.91	2.41	3.185 (11)	144
N3—H3*E*⋯O5^i^	0.91	1.90	2.766 (12)	157
O4—H4⋯Cl5	0.82	2.48	3.110 (9)	134
N4—H4*A*⋯O5	0.89	1.92	2.804 (12)	171
N4—H4*C*⋯Cl5^ii^	0.89	2.44	3.111 (11)	132
O5—H5*A*⋯Cl8	0.87	2.32	3.156 (10)	161
O5—H5*B*⋯Cl3^iii^	0.87	2.36	3.192 (10)	160

Symmetry codes: (i) 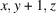 ; (ii) 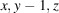 ; (iii) 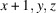 .

**Table 2:** Experimental details

Crystal data	
Chemical formula	(C_9_H_12_NO)_2_[PdCl_4_]·0.5H_2_O
*M*_r_	557.62
Crystal system, space group	Monoclinic, *P*2_1_
Temperature (K)	100
*a*, *b*, *c* (Å)	8.4593 (2), 8.3940 (2), 30.7294 (6)
β (°)	97.033 (1)
*V* (Å^3^)	2165.60 (8)
*Z*	4
Radiation type	Mo *K*α
μ (mm^−1^)	1.37
Crystal size (mm)	0.1 × 0.1 × 0.1

Data collection	
Diffractometer	Bruker APEXII CCD
Absorption correction	Multi-scan (*SADABS*; Bruker, 2014 ▸)
*T*_min_, *T*_max_	0.631, 0.745
No. of measured, independent and observed [*I* > 2σ(*I*)] reflections	29908, 8256, 5555
*R*_int_	0.096
(sin θ/λ)_max_ (Å^−1^)	0.615

Refinement	
*R*[*F* ^2^ > 2σ(*F* ^2^)], *wR*(*F* ^2^), *S*	0.057, 0.129, 1.05
No. of reflections	8256
No. of parameters	500
No. of restraints	2
H-atom treatment	H-atom parameters constrained
Δρ_max_, Δρ_min_ (e Å^−3^)	0.66, −1.75
Absolute structure	Flack *x* determined using 1805 quotients [(*I* ^+^)−(*I* ^−^)]/[(*I* ^+^)+(*I* ^−^)] (Parsons *et al.*, 2013 ▸)
Absolute structure parameter	−0.02 (3)

Computer programs: *APEX2* and *SAINT* (Bruker, 2014 ▸), (Bruker, 2014 ▸), *SHELXS1997* (Sheldrick, 2008 ▸), *XL* (Sheldrick, 2008 ▸) and *OLEX2* (Dolomanov *et al.*, 2009 ▸).

## full crystallographic data

### Crystal data

**Table:**

(C_9_H_12_NO)_2_[PdCl_4_]·0.5H_2_O	*F*(000) = 1124
*M**_r_* = 557.62	*D*_x_ = 1.710 Mg m^−^^3^
Monoclinic, *P*2_1_	Mo *K*α radiation, λ = 0.71073 Å
*a* = 8.4593 (2) Å	Cell parameters from 6451 reflections
*b* = 8.3940 (2) Å	θ = 3.4–25.8°
*c* = 30.7294 (6) Å	µ = 1.37 mm^−^^1^
β = 97.033 (1)°	*T* = 100 K
*V* = 2165.60 (8) Å^3^	Block, light yellow
*Z* = 4	0.1 × 0.1 × 0.1 mm

### Data collection

**Table:**

Bruker APEXII CCD diffractometer	5555 reflections with *I* > 2σ(*I*)
φ and ω scans	*R*_int_ = 0.096
Absorption correction: multi-scan (SADABS; Bruker, 2014)	θ_max_ = 25.9°, θ_min_ = 0.7°
*T*_min_ = 0.631, *T*_max_ = 0.745	*h* = −10→10
29908 measured reflections	*k* = −10→10
8256 independent reflections	*l* = −37→37

### Refinement

**Table:**

Refinement on *F*^2^	Hydrogen site location: mixed
Least-squares matrix: full	H-atom parameters constrained
*R*[*F*^2^ > 2σ(*F*^2^)] = 0.057	*w* = 1/[σ^2^(*F*_o_^2^) + (0.0454*P*)^2^ + 0.6412*P*] where *P* = (*F*_o_^2^ + 2*F*_c_^2^)/3
*wR*(*F*^2^) = 0.129	(Δ/σ)_max_ = 0.001
*S* = 1.05	Δρ_max_ = 0.66 e Å^−^^3^
8256 reflections	Δρ_min_ = −1.75 e Å^−^^3^
500 parameters	Absolute structure: Flack *x* determined using 1805 quotients [(I+)-(I-)]/[(I+)+(I-)] (Parsons *et al.*, 2013)
2 restraints	Absolute structure parameter: −0.02 (3)
Primary atom site location: structure-invariant direct methods	

### Special details

**Table:**

Geometry. All esds (except the esd in the dihedral angle between two l.s. planes) are estimated using the full covariance matrix. The cell esds are taken into account individually in the estimation of esds in distances, angles and torsion angles; correlations between esds in cell parameters are only used when they are defined by crystal symmetry. An approximate (isotropic) treatment of cell esds is used for estimating esds involving l.s. planes.

### Fractional atomic coordinates and isotropic or equivalent isotropic displacement parameters (Å^2^)

**Table:**

	*x*	*y*	*z*	*U*_iso_*/*U*_eq_	
Pd1	0.51806 (11)	0.01645 (12)	0.26089 (3)	0.0194 (3)	
Cl1	0.6917 (4)	0.2318 (4)	0.26513 (10)	0.0243 (8)	
Cl2	0.5878 (4)	−0.0454 (4)	0.33361 (10)	0.0298 (8)	
Cl3	0.3496 (4)	−0.2011 (4)	0.25398 (11)	0.0328 (8)	
Cl4	0.4437 (4)	0.0790 (4)	0.18830 (9)	0.0229 (7)	
Pd2	0.98172 (11)	0.53371 (12)	0.24203 (3)	0.0212 (3)	
Cl5	1.0264 (4)	0.6271 (4)	0.31266 (10)	0.0238 (8)	
Cl6	0.7552 (4)	0.6893 (4)	0.23258 (11)	0.0280 (8)	
Cl7	0.9281 (4)	0.4289 (4)	0.17266 (10)	0.0350 (9)	
Cl8	1.2149 (4)	0.3883 (4)	0.25495 (10)	0.0279 (8)	
O1	0.3933 (9)	0.7398 (10)	0.1478 (3)	0.028 (2)	
H1	0.3792	0.7855	0.1705	0.033*	
N1	0.4767 (11)	0.4627 (12)	0.1906 (3)	0.021 (2)	
H1A	0.4235	0.4902	0.2127	0.026*	
H1B	0.5533	0.5336	0.1881	0.026*	
H1C	0.5199	0.3668	0.1957	0.026*	
C1	0.3645 (14)	0.4583 (15)	0.1491 (4)	0.020 (3)	
H1D	0.2819	0.3750	0.1519	0.024*	
C2	0.2814 (15)	0.6179 (14)	0.1392 (4)	0.024 (3)	
H2	0.1888	0.6320	0.1562	0.029*	
C3	0.2294 (14)	0.6100 (16)	0.0904 (4)	0.030 (3)	
H3A	0.2231	0.7177	0.0771	0.036*	
H3B	0.1246	0.5569	0.0839	0.036*	
C4	0.3607 (13)	0.5117 (18)	0.0736 (4)	0.022 (3)	
C5	0.4035 (16)	0.4977 (19)	0.0316 (4)	0.032 (4)	
H5	0.3465	0.5538	0.0078	0.038*	
C6	0.5329 (16)	0.3988 (17)	0.0249 (4)	0.034 (4)	
H6	0.5631	0.3862	−0.0037	0.041*	
C7	0.6149 (17)	0.3211 (17)	0.0597 (4)	0.036 (4)	
H7	0.7044	0.2583	0.0548	0.043*	
C8	0.5722 (14)	0.3301 (15)	0.1026 (4)	0.023 (3)	
H8	0.6297	0.2740	0.1263	0.028*	
C9	0.4412 (14)	0.4256 (15)	0.1085 (4)	0.021 (3)	
O2	0.8692 (9)	0.4604 (10)	0.3926 (3)	0.030 (2)	
H2A	0.9532	0.4234	0.3863	0.037*	
N2	0.6483 (12)	0.5424 (15)	0.3288 (3)	0.032 (3)	
H2B	0.7439	0.5805	0.3390	0.039*	
H2C	0.5783	0.6220	0.3255	0.039*	
H2D	0.6532	0.4955	0.3030	0.039*	
C10	0.5966 (14)	0.4227 (15)	0.3603 (4)	0.022 (3)	
H10	0.5288	0.3392	0.3441	0.027*	
C11	0.7492 (14)	0.3447 (15)	0.3858 (4)	0.023 (3)	
H11	0.7853	0.2514	0.3693	0.027*	
C12	0.6896 (14)	0.2911 (17)	0.4286 (4)	0.025 (3)	
H12A	0.6416	0.1835	0.4256	0.030*	
H12B	0.7771	0.2906	0.4531	0.030*	
C13	0.5671 (14)	0.4145 (16)	0.4354 (4)	0.025 (3)	
C14	0.4972 (15)	0.4543 (16)	0.4728 (4)	0.030 (3)	
H14	0.5349	0.4068	0.5001	0.036*	
C15	0.3710 (15)	0.5644 (17)	0.4701 (4)	0.033 (4)	
H15	0.3211	0.5896	0.4953	0.039*	
C16	0.3206 (16)	0.6359 (17)	0.4298 (5)	0.037 (4)	
H16	0.2360	0.7110	0.4281	0.045*	
C17	0.3903 (16)	0.6011 (17)	0.3920 (4)	0.035 (4)	
H17	0.3549	0.6506	0.3648	0.041*	
C18	0.5127 (14)	0.4917 (16)	0.3961 (4)	0.025 (3)	
O3	0.5973 (9)	0.9031 (10)	0.0991 (3)	0.028 (2)	
H3	0.5198	0.8789	0.1114	0.034*	
N3	0.8073 (11)	0.9780 (13)	0.1686 (3)	0.026 (3)	
H3C	0.7524	0.9245	0.1877	0.031*	
H3D	0.7429	1.0525	0.1541	0.031*	
H3E	0.8932	1.0268	0.1837	0.031*	
C19	0.8619 (14)	0.8638 (15)	0.1366 (4)	0.021 (3)	
H19	0.9273	0.7788	0.1529	0.025*	
C20	0.7187 (14)	0.7862 (15)	0.1089 (4)	0.023 (3)	
H20	0.6795	0.6896	0.1233	0.028*	
C21	0.7854 (14)	0.7463 (16)	0.0656 (4)	0.028 (3)	
H21A	0.8322	0.6381	0.0668	0.034*	
H21B	0.7005	0.7523	0.0405	0.034*	
C22	0.9131 (14)	0.8726 (15)	0.0617 (4)	0.021 (3)	
C23	0.9887 (14)	0.9149 (15)	0.0265 (4)	0.026 (3)	
H23	0.9594	0.8686	−0.0015	0.031*	
C24	1.1095 (14)	1.0275 (19)	0.0330 (4)	0.029 (3)	
H24	1.1621	1.0600	0.0089	0.034*	
C25	1.1545 (16)	1.0925 (17)	0.0734 (4)	0.033 (3)	
H25	1.2404	1.1662	0.0771	0.039*	
C26	1.0770 (14)	1.0527 (15)	0.1091 (4)	0.025 (3)	
H26	1.1043	1.1021	0.1368	0.030*	
C27	0.9577 (13)	0.9380 (16)	0.1029 (4)	0.020 (3)	
O4	1.0775 (9)	0.2777 (10)	0.3450 (3)	0.028 (2)	
H4	1.1178	0.3539	0.3338	0.033*	
N4	0.9755 (10)	−0.0059 (12)	0.3100 (3)	0.024 (3)	
H4A	1.0166	0.0122	0.2852	0.028*	
H4B	0.9059	0.0706	0.3141	0.028*	
H4C	0.9263	−0.0997	0.3084	0.028*	
C28	1.1051 (13)	−0.0067 (15)	0.3470 (3)	0.021 (3)	
H28	1.1828	−0.0927	0.3421	0.025*	
C29	1.1930 (13)	0.1520 (15)	0.3522 (4)	0.024 (3)	
H29	1.2766	0.1605	0.3320	0.029*	
C30	1.2655 (15)	0.1539 (16)	0.4003 (4)	0.033 (4)	
H30A	1.3707	0.1006	0.4043	0.040*	
H30B	1.2773	0.2641	0.4117	0.040*	
C31	1.1432 (14)	0.0609 (17)	0.4228 (4)	0.028 (3)	
C32	1.1172 (16)	0.053 (2)	0.4668 (4)	0.038 (4)	
H32	1.1828	0.1120	0.4883	0.046*	
C33	0.9965 (17)	−0.0411 (19)	0.4788 (4)	0.041 (4)	
H33	0.9770	−0.0446	0.5086	0.050*	
C34	0.9044 (16)	−0.1296 (18)	0.4479 (4)	0.036 (4)	
H34	0.8214	−0.1942	0.4565	0.044*	
C35	0.9302 (15)	−0.1262 (16)	0.4049 (4)	0.031 (3)	
H35	0.8662	−0.1898	0.3841	0.037*	
C36	1.0479 (13)	−0.0315 (14)	0.3914 (4)	0.020 (3)	
O5	1.0720 (9)	0.0508 (12)	0.2273 (3)	0.033 (2)	
H5A	1.1084	0.1478	0.2282	0.049*	
H5B	1.1490	−0.0053	0.2410	0.049*	

### Atomic displacement parameters (Å^2^)

**Table:**

	*U*^11^	*U*^22^	*U*^33^	*U*^12^	*U*^13^	*U*^23^
Pd1	0.0202 (5)	0.0185 (6)	0.0201 (5)	−0.0019 (5)	0.0050 (4)	−0.0014 (5)
Cl1	0.0241 (16)	0.0201 (18)	0.0277 (19)	−0.0025 (14)	−0.0010 (14)	0.0005 (14)
Cl2	0.0345 (19)	0.029 (2)	0.0268 (18)	−0.0015 (15)	0.0061 (15)	0.0040 (15)
Cl3	0.0336 (19)	0.025 (2)	0.041 (2)	−0.0093 (16)	0.0104 (16)	−0.0034 (16)
Cl4	0.0275 (17)	0.0187 (18)	0.0219 (17)	−0.0005 (13)	0.0014 (14)	−0.0021 (13)
Pd2	0.0214 (5)	0.0224 (7)	0.0207 (5)	−0.0002 (5)	0.0060 (4)	0.0007 (5)
Cl5	0.0254 (17)	0.0215 (19)	0.0245 (18)	0.0003 (14)	0.0036 (14)	−0.0021 (14)
Cl6	0.0222 (17)	0.030 (2)	0.0323 (19)	0.0022 (15)	0.0042 (14)	0.0030 (15)
Cl7	0.038 (2)	0.044 (2)	0.0233 (19)	−0.0047 (18)	0.0051 (16)	−0.0054 (16)
Cl8	0.0275 (18)	0.0255 (19)	0.033 (2)	0.0018 (15)	0.0115 (15)	0.0010 (15)
O1	0.032 (5)	0.025 (5)	0.027 (5)	−0.012 (4)	0.008 (4)	−0.011 (4)
N1	0.028 (6)	0.019 (6)	0.018 (6)	0.000 (5)	0.006 (5)	−0.002 (4)
C1	0.019 (7)	0.021 (8)	0.019 (7)	−0.009 (6)	0.001 (5)	−0.008 (6)
C2	0.031 (8)	0.012 (7)	0.028 (8)	0.002 (6)	0.003 (6)	0.001 (6)
C3	0.020 (7)	0.018 (8)	0.051 (9)	0.008 (6)	0.003 (6)	0.005 (6)
C4	0.023 (7)	0.019 (7)	0.023 (7)	−0.008 (7)	−0.002 (5)	0.000 (7)
C5	0.041 (8)	0.034 (11)	0.019 (7)	−0.009 (8)	−0.004 (6)	0.003 (7)
C6	0.044 (9)	0.041 (9)	0.016 (7)	−0.012 (8)	0.002 (7)	−0.007 (7)
C7	0.055 (10)	0.028 (9)	0.025 (8)	0.002 (7)	0.009 (7)	−0.018 (7)
C8	0.026 (7)	0.018 (8)	0.025 (8)	−0.007 (6)	0.004 (6)	−0.002 (6)
C9	0.031 (8)	0.016 (7)	0.016 (7)	−0.005 (6)	0.001 (6)	−0.008 (5)
O2	0.024 (5)	0.030 (6)	0.038 (6)	−0.005 (4)	0.004 (4)	0.002 (4)
N2	0.040 (7)	0.027 (7)	0.031 (6)	−0.005 (6)	0.010 (5)	0.007 (6)
C10	0.031 (8)	0.013 (7)	0.025 (7)	0.001 (6)	0.012 (6)	0.000 (5)
C11	0.024 (7)	0.016 (7)	0.028 (8)	0.004 (6)	0.007 (6)	−0.001 (6)
C12	0.020 (7)	0.031 (8)	0.026 (8)	0.013 (6)	0.008 (6)	0.007 (6)
C13	0.030 (8)	0.029 (8)	0.018 (7)	−0.011 (7)	0.005 (6)	−0.001 (6)
C14	0.031 (8)	0.027 (8)	0.032 (8)	−0.007 (7)	0.004 (6)	0.001 (6)
C15	0.035 (8)	0.040 (11)	0.027 (8)	−0.001 (7)	0.019 (6)	−0.014 (7)
C16	0.031 (8)	0.031 (9)	0.052 (10)	0.013 (7)	0.019 (7)	−0.001 (7)
C17	0.040 (9)	0.032 (9)	0.032 (8)	0.001 (7)	0.010 (7)	0.004 (7)
C18	0.016 (6)	0.030 (9)	0.030 (7)	0.001 (6)	0.012 (6)	−0.014 (6)
O3	0.019 (5)	0.035 (6)	0.030 (5)	0.005 (4)	0.005 (4)	0.005 (4)
N3	0.025 (6)	0.038 (8)	0.015 (5)	0.006 (5)	0.005 (4)	−0.003 (5)
C19	0.023 (7)	0.021 (8)	0.019 (7)	0.005 (6)	0.000 (6)	0.004 (6)
C20	0.026 (7)	0.020 (7)	0.025 (7)	−0.003 (6)	0.010 (6)	0.004 (6)
C21	0.024 (7)	0.022 (8)	0.037 (9)	0.001 (6)	0.000 (6)	−0.011 (6)
C22	0.024 (7)	0.018 (7)	0.019 (7)	−0.004 (6)	−0.003 (5)	−0.001 (5)
C23	0.030 (7)	0.032 (9)	0.015 (7)	0.007 (7)	0.003 (6)	0.006 (6)
C24	0.035 (7)	0.025 (8)	0.028 (7)	−0.006 (8)	0.015 (6)	0.012 (7)
C25	0.033 (8)	0.030 (8)	0.037 (9)	−0.014 (7)	0.010 (7)	−0.002 (7)
C26	0.026 (7)	0.016 (8)	0.034 (7)	−0.004 (6)	0.004 (6)	−0.001 (6)
C27	0.011 (6)	0.027 (8)	0.025 (7)	0.008 (6)	0.005 (5)	0.001 (6)
O4	0.020 (5)	0.016 (5)	0.049 (6)	0.001 (4)	0.014 (4)	0.008 (4)
N4	0.023 (5)	0.022 (7)	0.027 (6)	−0.002 (5)	0.006 (4)	0.007 (5)
C28	0.020 (6)	0.020 (8)	0.020 (7)	−0.001 (6)	−0.010 (5)	0.005 (5)
C29	0.011 (6)	0.017 (8)	0.045 (9)	−0.006 (6)	0.008 (6)	−0.009 (6)
C30	0.027 (8)	0.020 (8)	0.052 (10)	0.001 (6)	0.002 (7)	−0.011 (7)
C31	0.019 (7)	0.023 (9)	0.042 (8)	0.009 (6)	0.002 (6)	−0.004 (7)
C32	0.054 (10)	0.036 (11)	0.021 (8)	0.005 (9)	−0.011 (7)	−0.014 (7)
C33	0.044 (9)	0.059 (11)	0.024 (8)	0.015 (8)	0.015 (7)	0.005 (7)
C34	0.033 (8)	0.048 (10)	0.030 (9)	0.012 (7)	0.011 (7)	0.019 (8)
C35	0.033 (8)	0.024 (8)	0.033 (8)	−0.010 (7)	−0.010 (7)	0.012 (6)
C36	0.016 (6)	0.014 (7)	0.029 (7)	0.005 (5)	0.001 (6)	0.007 (5)
O5	0.024 (5)	0.037 (6)	0.038 (5)	−0.006 (5)	0.006 (4)	−0.005 (5)

### Geometric parameters (Å, º)

**Table:**

Pd1—Cl1	2.323 (3)	C16—C17	1.397 (17)
Pd1—Cl2	2.300 (3)	C17—H17	0.9500
Pd1—Cl3	2.310 (3)	C17—C18	1.379 (17)
Pd1—Cl4	2.303 (3)	O3—H3	0.8205
Pd2—Cl5	2.295 (3)	O3—C20	1.425 (14)
Pd2—Cl6	2.307 (3)	N3—H3C	0.9100
Pd2—Cl7	2.299 (3)	N3—H3D	0.9100
Pd2—Cl8	2.312 (3)	N3—H3E	0.9100
O1—H1	0.8198	N3—C19	1.487 (14)
O1—C2	1.397 (14)	C19—H19	1.0000
N1—H1A	0.8897	C19—C20	1.536 (16)
N1—H1B	0.8901	C19—C27	1.523 (16)
N1—H1C	0.8899	C20—H20	1.0000
N1—C1	1.494 (13)	C20—C21	1.544 (16)
C1—H1D	1.0000	C21—H21A	0.9900
C1—C2	1.526 (16)	C21—H21B	0.9900
C1—C9	1.499 (15)	C21—C22	1.529 (16)
C2—H2	1.0000	C22—C23	1.369 (16)
C2—C3	1.514 (16)	C22—C27	1.389 (16)
C3—H3A	0.9900	C23—H23	0.9500
C3—H3B	0.9900	C23—C24	1.389 (17)
C3—C4	1.522 (17)	C24—H24	0.9500
C4—C5	1.389 (15)	C24—C25	1.366 (17)
C4—C9	1.399 (16)	C25—H25	0.9500
C5—H5	0.9500	C25—C26	1.386 (16)
C5—C6	1.410 (18)	C26—H26	0.9500
C6—H6	0.9500	C26—C27	1.390 (16)
C6—C7	1.366 (18)	O4—H4	0.8203
C7—H7	0.9500	O4—C29	1.437 (13)
C7—C8	1.411 (17)	N4—H4A	0.8896
C8—H8	0.9500	N4—H4B	0.8900
C8—C9	1.398 (16)	N4—H4C	0.8898
O2—H2A	0.8203	N4—C28	1.481 (13)
O2—C11	1.402 (14)	C28—H28	1.0000
N2—H2B	0.8899	C28—C29	1.524 (16)
N2—H2C	0.8902	C28—C36	1.516 (15)
N2—H2D	0.8899	C29—H29	1.0000
N2—C10	1.498 (15)	C29—C30	1.530 (17)
C10—H10	1.0000	C30—H30A	0.9900
C10—C11	1.568 (16)	C30—H30B	0.9900
C10—C18	1.495 (15)	C30—C31	1.527 (17)
C11—H11	1.0000	C31—C32	1.396 (17)
C11—C12	1.532 (16)	C31—C36	1.411 (17)
C12—H12A	0.9900	C32—H32	0.9500
C12—H12B	0.9900	C32—C33	1.376 (19)
C12—C13	1.498 (17)	C33—H33	0.9500
C13—C14	1.394 (16)	C33—C34	1.371 (19)
C13—C18	1.400 (17)	C34—H34	0.9500
C14—H14	0.9500	C34—C35	1.365 (17)
C14—C15	1.407 (17)	C35—H35	0.9500
C15—H15	0.9500	C35—C36	1.377 (16)
C15—C16	1.394 (17)	O5—H5A	0.8701
C16—H16	0.9500	O5—H5B	0.8700
			
Cl2—Pd1—Cl1	92.07 (11)	C18—C17—C16	117.0 (13)
Cl2—Pd1—Cl3	89.58 (13)	C18—C17—H17	121.5
Cl2—Pd1—Cl4	179.00 (12)	C13—C18—C10	108.7 (11)
Cl3—Pd1—Cl1	177.56 (13)	C17—C18—C10	127.7 (12)
Cl4—Pd1—Cl1	88.45 (12)	C17—C18—C13	123.4 (11)
Cl4—Pd1—Cl3	89.94 (12)	C20—O3—H3	109.2
Cl5—Pd2—Cl6	87.93 (12)	H3C—N3—H3D	109.5
Cl5—Pd2—Cl7	176.74 (13)	H3C—N3—H3E	109.5
Cl5—Pd2—Cl8	88.82 (11)	H3D—N3—H3E	109.5
Cl6—Pd2—Cl8	176.45 (13)	C19—N3—H3C	109.5
Cl7—Pd2—Cl6	91.92 (12)	C19—N3—H3D	109.5
Cl7—Pd2—Cl8	91.40 (12)	C19—N3—H3E	109.5
C2—O1—H1	109.7	N3—C19—H19	109.0
H1A—N1—H1B	109.5	N3—C19—C20	110.6 (9)
H1A—N1—H1C	109.5	N3—C19—C27	114.9 (10)
H1B—N1—H1C	109.5	C20—C19—H19	109.0
C1—N1—H1A	109.3	C27—C19—H19	109.0
C1—N1—H1B	109.6	C27—C19—C20	104.3 (10)
C1—N1—H1C	109.5	O3—C20—C19	108.9 (10)
N1—C1—H1D	108.8	O3—C20—H20	112.4
N1—C1—C2	112.2 (9)	O3—C20—C21	107.3 (9)
N1—C1—C9	114.8 (10)	C19—C20—H20	112.4
C2—C1—H1D	108.8	C19—C20—C21	102.8 (9)
C9—C1—H1D	108.8	C21—C20—H20	112.4
C9—C1—C2	103.4 (10)	C20—C21—H21A	110.8
O1—C2—C1	108.7 (9)	C20—C21—H21B	110.8
O1—C2—H2	111.8	H21A—C21—H21B	108.9
O1—C2—C3	109.0 (10)	C22—C21—C20	104.7 (10)
C1—C2—H2	111.8	C22—C21—H21A	110.8
C3—C2—C1	103.3 (10)	C22—C21—H21B	110.8
C3—C2—H2	111.8	C23—C22—C21	130.2 (11)
C2—C3—H3A	111.2	C23—C22—C27	121.1 (12)
C2—C3—H3B	111.2	C27—C22—C21	108.5 (10)
C2—C3—C4	102.9 (9)	C22—C23—H23	121.0
H3A—C3—H3B	109.1	C22—C23—C24	118.0 (12)
C4—C3—H3A	111.2	C24—C23—H23	121.0
C4—C3—H3B	111.2	C23—C24—H24	119.3
C5—C4—C3	130.1 (12)	C25—C24—C23	121.4 (11)
C5—C4—C9	120.8 (13)	C25—C24—H24	119.3
C9—C4—C3	109.2 (10)	C24—C25—H25	119.5
C4—C5—H5	120.6	C24—C25—C26	121.1 (12)
C4—C5—C6	118.8 (12)	C26—C25—H25	119.5
C6—C5—H5	120.6	C25—C26—H26	121.2
C5—C6—H6	120.2	C25—C26—C27	117.7 (12)
C7—C6—C5	119.7 (12)	C27—C26—H26	121.2
C7—C6—H6	120.2	C22—C27—C19	110.4 (11)
C6—C7—H7	118.6	C22—C27—C26	120.7 (11)
C6—C7—C8	122.8 (13)	C26—C27—C19	128.9 (11)
C8—C7—H7	118.6	C29—O4—H4	109.1
C7—C8—H8	121.6	H4A—N4—H4B	109.5
C9—C8—C7	116.8 (12)	H4A—N4—H4C	109.5
C9—C8—H8	121.6	H4B—N4—H4C	109.5
C4—C9—C1	108.9 (11)	C28—N4—H4A	109.2
C8—C9—C1	130.1 (11)	C28—N4—H4B	109.6
C8—C9—C4	121.0 (11)	C28—N4—H4C	109.6
C11—O2—H2A	109.5	N4—C28—H28	109.0
H2B—N2—H2C	109.5	N4—C28—C29	112.5 (9)
H2B—N2—H2D	109.5	N4—C28—C36	113.8 (9)
H2C—N2—H2D	109.5	C29—C28—H28	109.0
C10—N2—H2B	109.7	C36—C28—H28	109.0
C10—N2—H2C	109.6	C36—C28—C29	103.4 (10)
C10—N2—H2D	109.2	O4—C29—C28	108.1 (9)
N2—C10—H10	110.0	O4—C29—H29	111.8
N2—C10—C11	108.4 (9)	O4—C29—C30	109.1 (10)
C11—C10—H10	110.0	C28—C29—H29	111.8
C18—C10—N2	114.7 (10)	C28—C29—C30	104.0 (10)
C18—C10—H10	110.0	C30—C29—H29	111.8
C18—C10—C11	103.4 (10)	C29—C30—H30A	111.2
O2—C11—C10	108.8 (10)	C29—C30—H30B	111.2
O2—C11—H11	111.0	H30A—C30—H30B	109.2
O2—C11—C12	112.5 (10)	C31—C30—C29	102.6 (10)
C10—C11—H11	111.0	C31—C30—H30A	111.2
C12—C11—C10	102.3 (9)	C31—C30—H30B	111.2
C12—C11—H11	111.0	C32—C31—C30	131.2 (13)
C11—C12—H12A	111.2	C32—C31—C36	119.3 (13)
C11—C12—H12B	111.2	C36—C31—C30	109.5 (11)
H12A—C12—H12B	109.1	C31—C32—H32	120.1
C13—C12—C11	102.9 (10)	C33—C32—C31	119.9 (13)
C13—C12—H12A	111.2	C33—C32—H32	120.1
C13—C12—H12B	111.2	C32—C33—H33	119.9
C14—C13—C12	130.7 (12)	C34—C33—C32	120.1 (13)
C14—C13—C18	118.2 (12)	C34—C33—H33	119.9
C18—C13—C12	111.0 (11)	C33—C34—H34	119.5
C13—C14—H14	119.9	C35—C34—C33	121.0 (14)
C13—C14—C15	120.3 (12)	C35—C34—H34	119.5
C15—C14—H14	119.9	C34—C35—H35	119.7
C14—C15—H15	120.6	C34—C35—C36	120.7 (13)
C16—C15—C14	118.9 (11)	C36—C35—H35	119.7
C16—C15—H15	120.6	C31—C36—C28	108.7 (10)
C15—C16—H16	118.9	C35—C36—C28	132.2 (12)
C15—C16—C17	122.2 (13)	C35—C36—C31	119.0 (12)
C17—C16—H16	118.9	H5A—O5—H5B	104.5
C16—C17—H17	121.5		
			
O1—C2—C3—C4	82.6 (12)	O3—C20—C21—C22	85.6 (11)
N1—C1—C2—O1	42.7 (13)	N3—C19—C20—O3	38.7 (13)
N1—C1—C2—C3	158.4 (9)	N3—C19—C20—C21	152.3 (10)
N1—C1—C9—C4	−145.1 (10)	N3—C19—C27—C22	−138.8 (11)
N1—C1—C9—C8	34.0 (18)	N3—C19—C27—C26	42.4 (17)
C1—C2—C3—C4	−32.8 (12)	C19—C20—C21—C22	−29.2 (12)
C2—C1—C9—C4	−22.7 (13)	C20—C19—C27—C22	−17.6 (13)
C2—C1—C9—C8	156.4 (12)	C20—C19—C27—C26	163.6 (12)
C2—C3—C4—C5	−161.2 (14)	C20—C21—C22—C23	−165.7 (13)
C2—C3—C4—C9	19.9 (14)	C20—C21—C22—C27	19.5 (13)
C3—C4—C5—C6	179.2 (13)	C21—C22—C23—C24	−175.5 (12)
C3—C4—C9—C1	1.8 (14)	C21—C22—C27—C19	−1.2 (14)
C3—C4—C9—C8	−177.4 (11)	C21—C22—C27—C26	177.7 (11)
C4—C5—C6—C7	−1 (2)	C22—C23—C24—C25	1 (2)
C5—C4—C9—C1	−177.3 (11)	C23—C22—C27—C19	−176.6 (11)
C5—C4—C9—C8	3.5 (19)	C23—C22—C27—C26	2.3 (19)
C5—C6—C7—C8	2 (2)	C23—C24—C25—C26	−2 (2)
C6—C7—C8—C9	−0.9 (19)	C24—C25—C26—C27	3 (2)
C7—C8—C9—C1	178.9 (12)	C25—C26—C27—C19	175.4 (12)
C7—C8—C9—C4	−2.1 (17)	C25—C26—C27—C22	−3.3 (18)
C9—C1—C2—O1	−81.5 (11)	C27—C19—C20—O3	−85.3 (11)
C9—C1—C2—C3	34.2 (12)	C27—C19—C20—C21	28.3 (12)
C9—C4—C5—C6	−2 (2)	C27—C22—C23—C24	−1.2 (19)
O2—C11—C12—C13	84.6 (12)	O4—C29—C30—C31	83.0 (11)
N2—C10—C11—O2	35.4 (13)	N4—C28—C29—O4	40.6 (13)
N2—C10—C11—C12	154.6 (10)	N4—C28—C29—C30	156.4 (10)
N2—C10—C18—C13	−138.5 (11)	N4—C28—C36—C31	−143.9 (10)
N2—C10—C18—C17	47.6 (18)	N4—C28—C36—C35	38.1 (18)
C10—C11—C12—C13	−32.0 (12)	C28—C29—C30—C31	−32.2 (12)
C11—C10—C18—C13	−20.7 (13)	C29—C28—C36—C31	−21.6 (12)
C11—C10—C18—C17	165.4 (13)	C29—C28—C36—C35	160.4 (13)
C11—C12—C13—C14	−163.2 (13)	C29—C30—C31—C32	−162.0 (14)
C11—C12—C13—C18	20.9 (14)	C29—C30—C31—C36	19.6 (13)
C12—C13—C14—C15	−173.1 (13)	C30—C31—C32—C33	179.7 (13)
C12—C13—C18—C10	0.2 (15)	C30—C31—C36—C28	1.2 (14)
C12—C13—C18—C17	174.4 (12)	C30—C31—C36—C35	179.4 (11)
C13—C14—C15—C16	−1.8 (19)	C31—C32—C33—C34	2 (2)
C14—C13—C18—C10	−176.3 (11)	C32—C31—C36—C28	−177.5 (12)
C14—C13—C18—C17	−2 (2)	C32—C31—C36—C35	0.8 (19)
C14—C15—C16—C17	0 (2)	C32—C33—C34—C35	0 (2)
C15—C16—C17—C18	0 (2)	C33—C34—C35—C36	−1 (2)
C16—C17—C18—C10	173.9 (13)	C34—C35—C36—C28	178.5 (13)
C16—C17—C18—C13	1 (2)	C34—C35—C36—C31	0.7 (19)
C18—C10—C11—O2	−86.7 (11)	C36—C28—C29—O4	−82.7 (11)
C18—C10—C11—C12	32.5 (12)	C36—C28—C29—C30	33.2 (11)
C18—C13—C14—C15	2.5 (19)	C36—C31—C32—C33	−2 (2)

### Hydrogen-bond geometry (Å, º)

**Table:**

*D*—H···*A*	*D*—H	H···*A*	*D*···*A*	*D*—H···*A*
O1—H1···Cl4^i^	0.82	2.57	3.116 (9)	126
N1—H1*B*···Cl6	0.89	2.43	3.177 (10)	141
O2—H2*A*···O4	0.82	2.13	2.870 (11)	150
O3—H3···O1	0.82	2.01	2.779 (11)	155
N3—H3*C*···Cl6	0.91	2.41	3.185 (11)	144
N3—H3*E*···O5^i^	0.91	1.90	2.766 (12)	157
O4—H4···Cl5	0.82	2.48	3.110 (9)	134
N4—H4*A*···O5	0.89	1.92	2.804 (12)	171
N4—H4*C*···Cl5^ii^	0.89	2.44	3.111 (11)	132
O5—H5*A*···Cl8	0.87	2.32	3.156 (10)	161
O5—H5*B*···Cl3^iii^	0.87	2.36	3.192 (10)	160

Symmetry codes: (i) *x*, *y*+1, *z*; (ii) *x*, *y*−1, *z*; (iii) *x*+1, *y*, *z*.
